# How HIV-1 Gag Manipulates Its Host Cell Proteins: A Focus on Interactors of the Nucleocapsid Domain

**DOI:** 10.3390/v12080888

**Published:** 2020-08-13

**Authors:** Jéromine Klingler, Halina Anton, Eléonore Réal, Manon Zeiger, Christiane Moog, Yves Mély, Emmanuel Boutant

**Affiliations:** 1 INSERM UMR_S 1109, Centre de Recherche en Immunologie et Hématologie, Faculté de Médecine, Université de Strasbourg, 67000 Strasbourg, France; jeromine.klingler@gmail.com (J.K.); c.moog@unistra.fr (C.M.); 2 UMR 7021, CNRS, Laboratoire de Bioimagerie et Pathologies, Université de Strasbourg, Faculté de Pharmacie, 67400 Illkirch, France; halina.anton@unistra.fr (H.A.); eleonore.real@unistra.fr (E.R.); manon.zeiger@etu.unistra.fr (M.Z.); yves.mely@unistra.fr (Y.M.)

**Keywords:** HIV-1, Gag, Pr55Gag, interactants, NCp7, nucleocapsid

## Abstract

The human immunodeficiency virus (HIV-1) polyprotein Gag (Group-specific antigen) plays a central role in controlling the late phase of the viral lifecycle. Considered to be only a scaffolding protein for a long time, the structural protein Gag plays determinate and specific roles in HIV-1 replication. Indeed, via its different domains, Gag orchestrates the specific encapsidation of the genomic RNA, drives the formation of the viral particle by its auto-assembly (multimerization), binds multiple viral proteins, and interacts with a large number of cellular proteins that are needed for its functions from its translation location to the plasma membrane, where newly formed virions are released. Here, we review the interactions between HIV-1 Gag and 66 cellular proteins. Notably, we describe the techniques used to evidence these interactions, the different domains of Gag involved, and the implications of these interactions in the HIV-1 replication cycle. In the final part, we focus on the interactions involving the highly conserved nucleocapsid (NC) domain of Gag and detail the functions of the NC interactants along the viral lifecycle.

## 1. Introduction

**Figure 1:**
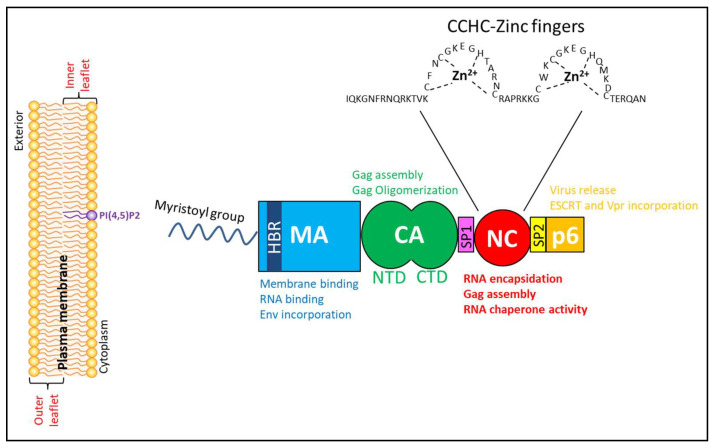
Schematic representation of the human immunodeficiency virus (HIV-1) Group-specific antigen (Gag) polyprotein, its different domains and their functions. HIV-1 Gag comprises different domains: matrix (MA), capsid (CA), nucleocapsid (NC), p6 and two spacer peptides (SP1 and SP2). The plasma membrane and phospholipid phosphatidylinositol 4,5-bisphosphate (PI(4,5)P2) located in the inner leaflet are indicated. Gag contains a myristoyl group at its N-terminal end (represented by the blue wave). The nucleocapsid (NC) is a characterized by two highly conserved zinc fingers separated by a basic linker.

The human immunodeficiency virus (HIV-1) genome encodes three major polyproteins: Gag (group-specific antigen), Pol (polymerase) and Env (envelope), two regulatory proteins: Tat (transactivator of transcription) and Rev (regulator of virion expression) as well as four accessory proteins: Vif (virion infectivity factor), Vpu (viral protein u), Vpr (viral protein r) and Nef (negative regulatory factor). In this review, we will focus on the Gag precursor that is synthesized in the cytoplasm as a multidomain protein of 55 kDa. Gag is composed of four major structural domains (from the N- to the C-terminus): MA (matrix), CA (capsid), NC (nucleocapsid) and p6 as well as two small peptides SP1 and SP2 (spacer peptides 1 and 2) linking respectively the CA-NC and NC-p6 domains (Figure 1). The mature Gag plays crucial functions in HIV-1 replication, notably during the assembly, budding, and release of infectious particles [1,2,3]. Each of its domains performs specific functions in the assembly process either alone or in collaboration with the other domains. The MA domain is implicated in the targeting and binding of Gag to the plasma membrane (PM). It also binds RNAs and mediates the incorporation of Env in virions. The CA domain mediates Gag multimerization. The NC domain is essential for the specific selection and packaging of genomic RNA (gRNA) inside virions and the stabilization of Gag oligomers. Finally, p6 is vital for the recruitment of the endosomal sorting complex required for transport (ESCRT) machinery, a mandatory step for effective budding from the PM, while SP1 seems to play a role in immature particle assembly. Therefore, the structural protein Gag is essential for the production of HIV-1 particles, which is further confirmed by the fact that its expression alone leads to the production of virus-like particles (VLPs). HIV-1 assembly takes place at the PM [4,5] or at a specific intracellular location named virus-containing compartment (VCC), which are PM invaginations in the cytoplasm, initially thought to be late endosomal structures in macrophages [5,6,7,8,9,10,11].

The viral budding and release are synchronized with the activation of the viral protease which cleaves Gag-Pol and Gag in a well-defined order and marks the onset of the maturation process. The successive cleavages of Gag polyprotein lead to particle rearrangements and dramatic modifications in the global virion morphology [12,13,14]. Cryo-electron microscopy, X-ray and nuclear magnetic resonance (NMR) studies enabled the complex process of the structural aspects of HIV-1 maturation to be deciphered. In the immature particles approximately 5000 copies of Gag molecules are aligned and form a hexagonal ordered lattice that adopts a wide range of curvatures and contains also non-ordered domains [15,16,17]. During the maturation this immature lattice disassembles, and CA molecules reassemble to form a fullerene capsid composed of a hexagonal lattice that contains 12 pentamers located at the ends of the capsid cone. MA remains associated to the viral membrane and the capsid shell protects tightly condensed viral genome coated with nucleocapsid and viral enzymes necessary for the viral replication. For more insight into the structural details of Gag polyprotein over the viral cycle and during the maturation see [3,16,18,19,20,21,22,23,24].

The role of Gag in viral assembly involves a set of interactions with a variety of different biomolecules, including different types of RNAs (transfer RNA (tRNA)), messenger RNA (mRNA)), gRNA), lipids, and viral and cellular proteins. In this review, these latter are specifically discussed in detail.

The first identification of the proteins present in HIV-1 virions took place in 1996 [25]. Ten years later, technology improvements with, notably, the use of liquid chromatography-linked tandem mass spectrometry (LC–MS/MS), allowed the identification of a large number of cellular proteins present in virions produced by monocyte-derived macrophages [26]. Between 2007 and 2009, multiple wide RNA interference (RNAi)-based screens were performed to identify HIV host factors involved only during the early [27] or during both the early and late stages [28,29,30] of the HIV-1 replicative cycle, as reviewed in Pache et al., 2011 [31]. To further characterize these putative interactions, several meta-analyses were performed [32,33]. In 2012, Jäger et al. used the technique of affinity purification/mass spectroscopy (AF/MS) to study the interaction of all viral proteins with cellular host factors from two different cell lines [34]. By compiling all these published data, the first global map of human protein complexes involved in HIV infection was established [35]. Several additional studies focused on HIV-1 Gag interacting factors using different technologies such as proximity-dependent biotinylation [36,37] and AF/MS screens [38]. It is also worth mentioning, that in some cases only a specific domain of Gag was used to identify specific host factors (for example, the MA domain [37,39]).

In this review, we will summarize the interactions between Gag and host cell factors, describing the domain of Gag implicated in the binding and the possible role, if identified, of the complex in the HIV-1 cell cycle. We will then focus on the interactions specifically occurring between Gag’s NC domain and cellular proteins and describe the putative functions of the complexes in the HIV-1 replication cycle. To our knowledge, this is the first exhaustive review of the interactions between Gag and cellular proteins.

## 2. Human Immunodeficiency Virus (HIV-1) Group-Specific Antigen (Gag) Structural Polyprotein

### 2.1. Matrix Domain of HIV-1 Gag

The matrix (MA) domain of Gag is composed of 132 amino acids and is post-translationally myristoylated at its N-terminus. Its functions in Gag assembly have been discussed in multiple reviews [1,2,40,41,42,43,44,45,46,47,48,49,50]. In summary, the MA domain of Gag participates in virus assembly through its ability to target and anchor Gag to the PM, via its myristoyl group (myr) and a highly basic region (HBR) (Figure 1). The myristoyl group could be sequestered into the MA domain to avoid aberrant binding, or solvent-exposed to favor its insertion into the cytoplasmic inner leaflet of the PM. The transition between these two states is termed the myristoyl (myr) switch [51,52,53,54,55,56]. Gag oligomerization is thought to promote myristate exposure [56], in line with the fact that multimeric MA possesses an increased affinity for the PM compared to monomeric MA [57]. The myr switch can be modulated by pH, revealing the MA domain as an in vitro pH sensor [58]. The HBR (located between amino acids 17 and 32 of MA) is rich in arginine and lysine residues and electrostatically interacts with the negatively charged phospholipids located in the inner leaflet of the PM [59,60,61]. This interaction of the MA domain of Gag with the PM seems to be mainly specific to the phospholipid phosphatidylinositol 4,5-bisphosphate (currently named PI(4,5)P2) [54,62,63,64,65,66]. This specific interaction was highlighted by the effect of phosphoinositide 5-phosphatase IV overexpression: an enzyme used to deplete cellular PI(4,5)P2. As a result of this overexpression, Gag was not correctly targeted to the PM but instead to late endosomes with a drastic effect on virus production [67]. Moreover, stimulation of PI(4,5)P2 production facilitated the accumulation of Gag in PI(4,5)P2 enriched intracellular vesicles, which resulted in reduced viral particles production [67]. Additional publications confirmed the interaction of PI(4,5)P2 with Gag and its essential role for efficient virus release in HeLa or T cells [64,68]. PI(4,5)P2 thus plays a dual role, by triggering the myristoyl switch and anchoring Gag at the PM [54] in an organized manner [62]. It is also important to note that cholesterol enhances the binding of Gag to the PM [62,69,70].

In addition, the same positively charged domain HBR is also able to bind RNA [40,63,71,72] (Figure 1). The role of RNA in HIV-1 assembly was already nicely reviewed [73,74]. Liposomes containing PI(4,5)P2 specifically compete with RNA for binding to MA [63] and ex vivo studies have shown that RNAs prevent the cytosolic Gag from binding to membranes containing phosphatidylserine (PS) [75]. RNA could thus prevent the binding of MA to a non-specific cell membrane that does not contain PI(4,5)P2. RNAs may also act by reducing myr-exposure [76]. Crosslinking and immunoprecipitation studies (CLIP) have shown that the MA domain selectively binds to tRNA and not viral RNA (vRNA) in infected cells. This binding to tRNA may regulate Gag binding to the PM [57,77], potentially inhibiting the assembly of genome-free viruses [78]. Using NMR and isothermal titration calorimetry experiments, Gaines et al. [79] studied specifically the effect of tRNA^lys3^ on Gag membrane targeting. They showed that by interacting with the conserved basic patch of MA, tRNA^Lys3^ could block the association of MA to liposomes including those enriched in PI(4,5)P2. In contrast, the myristoyl group exposure inhibits tRNA^Lys3^ binding to MA [79]. Recently, cell-imaging microscopy using MA-HBR mutants confirmed a key role of RNA on the specific PM targeting of Gag [80]. Of note, not all retroviruses respond in the same way to PI(4,5)P2 and RNA-mediated inhibition. Indeed, the binding of MA domains of HIV-1 and Rous sarcoma virus (RSV) Gag to the membranes is dependent on PI(4,5)P2 and is inhibited by RNA, but not in the cases of human T cell leukemia/lymphoma virus type 1 (HTLV-1), murine leukemia virus (MLV) or human endogenous retrovirus-K (HERV-K) Gag proteins [81]. Finally, the MA domain interacts with Env to facilitate its incorporation into virions [82,83] (Figure 1). Therefore, the MA domain not only ensures Gag targeting to the PM, allowing the budding of new virions, but also recruits viral (Env) and cellular partners that are essential for its function, as described in Table 1.

### 2.2. Capsid Domain of HIV-1 Gag

The interactions between the CA domains in Gag play a central role in the formation of the immature lattice found in the budding particle. The assembly of CA proteins into a fullerene-shaped structure composed of approximately 250 CA-hexamers and 12 CA-pentamers forms the viral capsid, a protective shell for the viral gRNA inside the mature particle and upon entry in the cell cytoplasm [84,85]. The viral capsid defines the frontier between the virus and the cell cytoplasm in the early phase of infection.

CA is an α-helical protein composed of two independent domains termed the N-terminal domain (CA-NTD) and the C-terminal domain (CA-CTD), which are separated by a short linker (Figure 1). CA-NTD is composed of 7 α-helices and a cyclophilin A (CypA)-binding loop with an N-terminus, which is unstructured in Gag but folded into a β-hairpin in the mature CA [86,87]. The CA-CTD is made up of a 3_10_ helix, a major homology region (MHR, highly conserved and essential for viral replication) loop and four α-helices. The C-terminus of CA-CTD undergoes an important structural rearrangement during virus maturation [88,89]. In immature virions, the junction between the C-terminus of the CA-CTD domain and SP1 forms a six-helix bundle within the Gag hexamer, after cleavage, the CA-SP1 junction becomes disordered [15]. The hexameric Gag lattice in the immature particle also depends on CA-CTD dimerization mediated by two amino acids: Trp 184 and Met 185. In the mature particle, in the CA hexamers, CA-NTD-CA-NTD and CA-NTD-CA-CTD contacts between adjacent monomers are found. Moreover, the hexamers interact with one or two other hexamers via the CA-CTD. The pentamers found at sites of high curvature expose different residues on the outer surface of the capsid relative to the hexamers, which could lead to different interactions with CA cellular partners [20,90]. The mechanism of mature lattice formation is still not fully understood. There is approximatively 2000 Gag per virion, but only around 1500 copies of CA are found in the mature lattice [16,91,92,93]. This large difference suggests that a large number of monomeric CA copies are released in the infected cells and may play a functional role [94]. The structural rearrangement of CA during the maturation are reviewed in [22,95].

CA is a structural protein that plays important roles in both the early and late phases of HIV-1 infection. In the cytoplasm, the viral core is initially found as a ribonucleoprotein containing the single-stranded (ss) gRNA (reverse transcription complex, RTC). During reverse transcription, the gRNA is converted into a double-stranded (ds) DNA, giving rise to the pre-integration complex (PIC) that is ready for genomic integration. During its conversion from RTC to PIC, the ribonucleoprotein traffics from the cell periphery to the nucleus and progressively releases the CA proteins. This uncoating mechanism and its precise timing have been debated for a long time. Currently, it is believed that a certain amount of CA proteins remains associated with the RTC/PIC even after its entry into the nucleus. CA proteins are thought to be key coordinators of several post-entry events. Their interaction with CypA encapsidated in the virions from the producing cells is necessary for HIV-1 to escape from the restriction factor TRIM5α found in the infected cells [96]. CA proteins are also implicated in the cytoplasmic traffic toward the nucleus, by interacting with the microtubule-associated proteins MAP1A and MAP1S, which tether the incoming viral CA to the microtubule network [97]. Moreover, by binding to fasciculation and elongation protein zeta 1 (FEZ1) and bicaudal D2 (BICD2), the adaptor proteins of kinesin-1 and dynein, respectively, CA proteins regulate the RTC/PIC movement in the cytoplasm [98,99,100]. CA proteins also participate in the nuclear import by interacting with Nup358/RanBP2, a nucleoporin that forms the cytoplasmic filaments of the nuclear pore. The interaction between CA proteins and Nup358/RanBP2 is instrumental for the RTC/PIC docking to the nuclear pore complexes (NPC) [101,102]. Another nucleoporin, Nup153, a component of the nuclear basket of the NPC, interacts with hexameric CA with much higher affinity than with CA monomers [103,104] and facilitates nuclear entry. This suggests that intact CA hexamers are associated with the RTC/PIC complex during nuclear import [105]. Furthermore, Nup153 plays a role in the selection of the integration site [106]. CPSF6, a predominantly nuclear component of the cleavage factor 1 (CFIm) complex, implicated in mRNA polyadenylation is also a hexameric CA binding protein [104,107]. CPSF6 is transported in the nucleus by binding to TNOP3 whose depletion leads to HIV-1 nuclear entry reduction. CPSF6-CA interaction is thought to regulate PIC intranuclear localization and direct HIV-1 integration to specific sites [108,109]. CA also binds Transportin-1, a β-karyopherin that facilitates the uncoating and the nuclear import of PIC [110].

CA thus seems to have more than a structural role, being a key element all along the viral cell cycle (Figure 1).

### 2.3. The Nucleocapsid (NC) Domain of HIV-1 Gag

The nucleocapsid domain of Gag (NC) and the corresponding mature form of the protein (NCp7) are basic polypeptides of 55 amino acids characterized by two conserved CCHC (Cys-X2-Cys-X4-His-X4-Cys) zinc fingers (ZFs) separated by a short linker and flanked by small domains rich in basic residues (Figure 1) [49,111]. The ZFs chelate zinc with a high affinity leading to their folding into a common fold while the other parts of NC are poorly structured. The folding of the ZFs allows the formation of a hydrophobic plateau that includes amino acids of the proximal (Val12, Phe16, Thr24 and Ala25) and the distal (Trp37, Gln45 and Met46) ZFs [112,113,114,115,116,117]. This plateau and the zinc binding residues play important roles in the virus-cell cycle as point mutations in these residues lead to non-infectious particles [45,118,119,120,121,122]. The ZFs and the hydrophobic plateau bind to the backbone and nucleobases of the nucleic acids (NAs). Moreover, the high flexibility of the protein allows it to bind to a large variety of NA sequences [123,124,125,126]. Interestingly, NCp7 exhibits different binding modes for RNA and DNA, and its affinity for ssNAs is higher than for dsNAs [112].

The importance of NC structure conservation explains its high sequence conservation in HIV-1 subtypes and isolates from treated patients and the low probability of mutations found in treatment-resistant strains [112,127].

The nucleocapsid is essential for several viral activities, which rely on its ability to bind NAs and to chaperone them. This NA chaperone activity allows the NAs to reach among their various possible conformations the most energetically and functionally favorable one [111,119]. This NA remodeling activity of the nucleocapsid relies on its NA destabilization and annealing properties [128,129,130,131,132,133,134,135,136,137,138]. As a result of their chaperone activity, NCp7 plays an important role in reverse transcription, where NCp7 enhances the reverse transcriptase (RT) processivity and RNase H activity [139,140] and in the integration step which is also stimulated by NCp7 [141]. The NC domain of Gag is the main actor for the selection of gRNA. This selection begins by the interaction of a few Gag proteins that preferentially bind to stem-loop sequences (SL1 and SL3) of the Psi region, resulting in the selection of the gRNA among the pool of cellular RNAs and its dimerization [45,49,74,77,142,143,144,145,146,147,148,149,150,151]. Currently, the precise location and timing of gRNA dimerization are controversial. Some studies support that dimerization is initiated in the cytosol [145] while others proposed that dimerization occurs preferentially at the PM [152]. After this nucleation step, Gag and Gag-Pol proteins accumulate on the gRNA via Gag-RNA, Gag–Gag and Gag–PM interactions, ultimately leading to a dimeric gRNA highly coated by Gag at the cell PM [119,131,153]. Different models for specific HIV-1 gRNA packaging were discussed in a recent review [49]. One of these models, based on in vitro conformational studies of Gag, suggests a key role for a U-shaped bent conformation of Gag, in which MA and NC are close together [154,155,156]. This conformation allows MA and NC to interact with the same binding partners [77,131,154,157]. Thus, during assembly, the MA and NC domains in the bent Gag conformation may simultaneously bind to gRNA and allow its transport through the cytoplasm [49,157,158]. This conformation could prevent MA from interacting with internal membranes; the HBR region being inaccessible to these membranes due to its binding to RNA [49,76]. Once Gag reaches the PM, the simultaneous presence of gRNA and PI(4,5)P2 causes MA and NC to interact with their preferential partner [49,131,154,156]. Therefore, NC remains linked to gRNA, while MA detaches from it to interact with PI(4,5)P2 at the PM, causing a conformational change that leads to the extended linear form of Gag [76,157,158].

During viral assembly, Gag-NC interacts with several cellular partners such as tRNA^Lys3^ [159], actin motors [160] and ESCRT machinery components in order to allow the budding of functional virions at the cell PM (Table 1). The NC domain is also the partner of cellular restriction factors such as APOBEC3G, a cytidine deaminase with an anti-HIV activity [161].

In addition to the specific selection of gRNA, NC interaction with NAs helps to promote Gag oligomerization in the cytoplasm. The two ZFs are important for Gag oligomerization (in addition to CA–CA interactions) and its traffic through the cytoplasm to reach the PM [162]. NC can also interact with negatively charged lipids in membranes, but, unlike MA, NC has no preference for PI(4,5)P2-containing membranes, such as the inner leaflet of the PM [157].

### 2.4. p6 Domain of HIV-1 Gag

Gag p6 is a multifunctional domain that is crucial in the late phase of the viral cycle (Figure 1). It corresponds to the 52 amino acids located at the C-terminus of the Gag polyprotein and contains several conserved motifs involved in the interactions with viral (Vpr) and cellular proteins (Tsg101, ALIX, ERK-2, aPKC). Its biological role is further tuned by post-translational modifications such as ubiquitination, SUMOylation and phosphorylation [163,164,165,166,167,168,169,170].

The p6 domain plays a critical role during the scission (pinching off) of the nascent virions from the cell surface by the cellular ESCRT machinery. Besides viral budding, the members of the ESCRT family are involved in various, topologically similar membrane remodeling processes such as the abscission step of cytokinesis, autophagy, wound-healing or exosome production (for review see [171,172,173,174]).

The ESCRT components bind specifically to the PTAP and LYPXnL sequences of p6, so-called late (L)-domains. Mutations in PTAP motif, located near the N-terminus of p6, [175,176] result in an inhibition (or a complete loss) of the viral release accompanied by default in Gag processing [164,177]. Electron microscopy (EM) images of cells expressing these PTAP mutants show numerous immature viral particles that remain attached to the PM or the membrane of intracytoplasmic vesicles [177].

PTAP recruits the ESCRT machinery by binding to the ubiquitin E2 variant (UEV) domain of the tumor susceptibility gene 101 (Tsg101) protein [164,177,178,179]. Tsg101 (44 kDa) is a member of the ESCRT I complex that mediates the sorting of ubiquitinated cargos into multivesicular bodies (MVBs). When bound to the PTAP site of Gag, Tsg101 activates the assembly of the ESCRT III complex that spirally polymerizes and shrinks the neck of the budding particle [180,181,182]. This polymerization activates VPS-4, an ATP free hydrolase, which in turn disassembles the ESCRT-III domain by hydrolyzing its proteins leading to the pinching off of the virion from the PM [172]. A recent study has reported that the proper recruitment of Tsg101 by PTAP is further controlled by RNA (probably bound to NC domain) and that the latter prevents Gag polyubiquitination and thus its degradation [183].

The second L-domain of the p6 protein, LYPXnL serves as a docking site for the apoptosis-linked gene 2-interacting protein X (ALIX/AIPI) [184,185,186,187,188]. ALIX (96 kDa) is a multifunctional adaptor protein that plays a key role in the regulation of intracellular protein trafficking and apoptosis. The function of ALIX in the release of HIV-1 requires the interaction of its N-terminal Bro 1 domain with the ESCRT-III components CHMP4B and the binding of its C-terminal proline-rich domain (PRD) to Tsg101 [186,187,189,190,191]. In normal conditions PTAP-Tsg101 is the main ESCRT activation pathway, but in PTAP-deleted HIV-1 mutants the budding phenotype can be completely rescued by the LYPXnL-ALIX pathway activated by ALIX overexpression [184,188].

A recent study depicting the HIV-release kinetics brought a new perspective on the role of ESCRT-p6 interactions in the release of infectious virions. Rather than a complete budding arrest, the L-domain mutations would induce a transient delay of the budding process which leads to a situation when the viral protease is activated before the VLP neck closure and in consequence the Pol products quit the viral particles and diffuse back into the cytosol. Interestingly the role p6 L-domains in the recruitment of ESCRT members is related to the size of “cargo” to be incorporated into the VLPs. Small cargoes rely mostly on PTAP/Tsg101 pathway, while bigger complexes require activation of both PTAP-Tsg101 and LYPXnL-ALIX pathways for the proper VLPs budding and release [192].

The p6 domain of Gag is also critical for the packaging of several viral proteins into the nascent virions. The best-known example is the p6 driven incorporation of Vpr, via its binding to ^41^LXXLF and ^15^FRFG sequences of p6 [193,194,195]. Vpr-p6 affinity is enhanced in a lipid environment [196], but PM anchoring is dispensable for their interaction [197]. The ^36^YPLTSL sequence plays a role for the incorporation of Env [72] and the proline rich C-terminal region of p6 drives the packaging of cleaved Pol proteins [198,199]. The role of the p6 central region is unclear and probably dispensable for p6 functions since its polymorphism does not perturb the viral infectivity nor the replication kinetics [200].

Finally, a recent study has revealed an unexpected role of p6 domain in the specific recognition and encapsidation of the viral gRNA [144].

### 2.5. Spacer Peptide (SP1) and SP2 Domains of HIV-1 Gag

The SP1 domain is composed of 14 amino acids and is located between the CA and the NC domains of Gag (Figure 1). The CA-SP1 region is a key regulator of Gag assembly. Mutations in the first seven SP1 residues, facing the CA domain, perturb the viral assembly and lead to the generation of tubular structures containing unprocessed Gag at the PM [201,202]. The SP1 domain may act as a switch for activation of Gag multimerization. At the early stages of the viral assembly, Gag oligomerization is thought to induce a conformational change of the CA-SP1 region from an unstructured coil state to a six α-helix bundle that favors Gag auto-assembly via SP1-SP1 interactions [201,202,203,204,205,206]. Mutation studies further suggest that the role of SP1 in Gag multimerization is also related to its membrane binding [207]. Recent structural and functional data have shown that the SP1 helix bundle is further stabilized by inositol hexakisphosphate (IP_6_). Upon protease cleavage, IP_6_ interactions also promote capsid maturation [208]. Thus, besides its role in Gag multimerization, the CA-SP1 cleavage site acts as a regulatory switch for the maturation of the HIV-1 particles. In addition, SP1 favors the recognition of gRNA Psi site by the NC domain [209,210,211]. It was shown that in the minimal Gag context, SP1 favors specific packaging of gRNA but not of spliced forms of vRNA [211,212].

The SP2 domain is composed of 16 amino acids and separates the NC and p6 Gag domains (Figure 1). The role of SP2 is less known. Mutagenesis studies suggest a possible implication of its proline residues in the processing of Gag polyprotein, the control of gRNA dimer stability and the packaging of Gag-Pol into nascent virions. These mutations also abolish the infectivity of multiple HIV-1 strains in peripheral blood monolayer cells [213]. While the proper processing between NC and p6 appears crucial for viral infectivity and maturation, the SP2 itself seems dispensable, and its deletion has only minor effect on the viral infectivity [214].

## 3. Interactions Between HIV-1 Gag Protein and Cellular Proteins

Although Gag is able to interact with HIV-1 viral proteins like Vpr, Vif, Env or itself, this review focuses on Gag-interacting cellular proteins which are listed and described in Table 1. Data presented in this table are as follows: cellular partner implicated in the interaction, classification of the interacting protein, the role of the protein, Gag domain involved in the interaction, the potential function of the complex during HIV-1 viral replication, experiments used to demonstrate the interaction and, finally, references related to the interaction.

## 4. Interactions Involving the NC Domain of Gag

**Figure 2:**
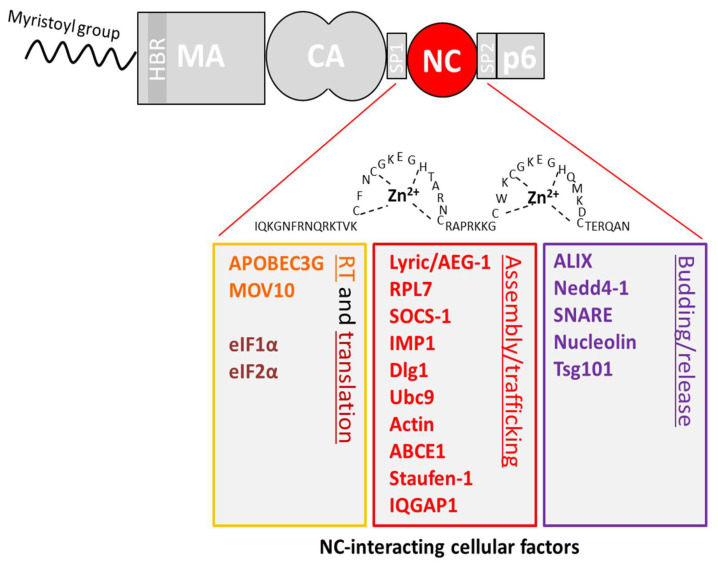
Zoom on the interaction of the NC domain of Gag with cellular host factors. The NC domain is highlighted, and its main cellular interacting partners are indicated.

The interactions of Gag NC domain with cellular proteins (Figure 2) will be presented according to their implication in different steps of the viral cycle (i.e., reverse transcription, translation, viral assembly or viral budding).

### 4.1. Reverse Transcription (RTion)

Thanks to its NAs chaperone activity, NCp7 is implicated in the RTion process by helping the NAs remodeling. In consequence, the minus-strand DNA exists for a shorter time as ssDNA substrate for the NC-binding restriction factor APOBEC3G (A3G) [139].

APOBEC proteins and especially A3G are incorporated in the virions via their interaction with the NC domain of Gag [161,239,240,241,324,325]. A3G is a host cytidine deaminase that restricts the replication of HIV-1 viruses lacking the viral protein Vif [326]. In the absence of Vif, A3G of the producing cell is incorporated into the virions during assembly by interacting with the NC domain of Gag. In the presence of Vif, A3G is targeted to the ubiquitination/proteasome pathway or inactivated, leading to the inhibition of its incorporation into the virion [327].

A3G binds to ssRNA and ssDNA with the same affinity even if the latter is the sole substrate of deamination. The A3G-induced C-to-U deamination of the ss minus-stranded DNA leads to G-to-A hypermutations in the vDNA positive strand, leading to a viral replication failure called error catastrophe. In parallel, the C-to-U hypermutation is thought to trigger the degradation of the reverse transcripts by cellular uracil DNA glycosylases (UDGs) [328]. Uracilated transcripts are also believed to show a decreased integration capacity [324,329].

In parallel, A3G also exerts a deamination-independent effect. Guo et al. [330,331] proposed that A3G can decrease the NC-mediated tRNA^Lys3^ annealing efficiency, but other groups did not observe this effect [332,333]. By binding to gRNA, A3G also reduces the rate of RT polymerization, by blocking RT movement along ssNAs with the same mechanism as for Gag. In contrast to NCp7, Gag and A3G associate and dissociate with NAs very slowly, leading to a block of RT movements along the RNA.

The human genome encodes seven members of the APOBEC3 family. Several of them interact with Vif (A3D, F, G and H) and restrict HIV-1 infection via deamination-dependent and/or independent process. Other members, such as A3A, also affect HIV infection but do not seem to interact with the NC domain of Gag, which explains why they are not incorporated in HIV virions [334].

The moloney leukemia virus 10 homolog (MOV10) is a member of the super-family-1 RNA helicase expressed in a variety of cell types and incorporated in HIV virions via its interaction with the NC domain of Gag [284,285]. MOV10, contrary to RHA, exhibits an inhibitory effect on HIV-1 infection independent of NC, which is attributed to the inhibition of RTion but is still debated [285,335,336]. MOV10 could act on HIV-1 infection by preventing A3G from Vif-induced proteasomal degradation leading to an increase in the A3G level. To do so, MOV10 could inhibit the formation of a complex around Vif, which induces A3G ubiquitination [337].

### 4.2. Translation

Cimarelli and colleagues [258] reported an interaction between the NC domain of Gag and the protein eEF1α, one of the two subunits of the eukaryotic translation elongation factor 1 (eEF1) involved in the elongation of the growing peptide chain. eEF1α is a GTP-bound protein that brings the aminoacylated tRNA to the elongating ribosome A site where the interaction with the anticodon occurs. This interaction is RNA dependent and occurs with both the MA and NC domains of Gag. Since both NC and eEF1α interact with tRNA [338] and NCp7 promotes tRNA annealing onto viral gRNA [339], this particular RNA could be involved. During the course of infection, Gag may interact with eEF1α-tRNA complex and thus inhibit translation elongation, which could lead to the dissociation of gRNA from the ribosome and the stimulation of its packaging into the virion. As eEF1α is incorporated into the virions and interacts with the actin skeleton as well as with components of the viral replication complex, eEF1α may have an active role in virion assembly and budding. Importantly, eEF1α also interacts with HIV RT, IN and Nef. In connection with its interaction with the RT, eEF1 is implicated in the late steps of RTion [340].

The NC domain of Gag is also acting on global host cell translation by modulating stress granule (SG) formation. Two types of SGs have been described that differ in their mechanism of assembly, localization, morphology, and composition. Type-I SGs such as those induced by sodium arsenite include eIF3 and promote cell survival, whereas type-II SGs are induced by selenite, or nitric oxide excludes eIF3, and enhance cell death. The formation of both types of SG depends on global translation inhibition. Both SGs contain stalled translation initiation complexes and thus a number of mRNA and eukaryotic initiation factors. Gag interacts via its CA domain with the eEF2 translation elongation factor and thus blocks the assembly of SG to the benefit of the formation of large detergent-insoluble HIV-1-dependent ribonucleoprotein complexes (SHRNP) containing the chaperone protein dsRNA-binding Staufen homolog 1 (Staufen-1, Stau1). SHRNPs are composed not only of Gag but also of other HIV-1 viral proteins and multiple cellular factors like Upf1 and IMP1 [308]. This SHRNP represents neither a SG nor a processing P-body (PB). Upon arsenic treatment (that stresses the cells), HIV-1 prevents the formation of SG in the cytoplasm and enhances SHRNP formation. By regulating the cellular machinery, HIV-1 could thus ensure a productive viral assembly under stress [302]. SHRNPs could be in equilibrium with active cell polysomes and thus, act on the balance between translation and vRNA encapsidation [302,303,305,308]. Finally, the team of Mouland confirmed the central role of Stau1 in HIV-1 replication. By using Stau1−/− gene-edited cells, this team demonstrated HIV-1 inability to dissociate SG with vRNA, leading to viral production and infectivity decrease [341].

On another side, NCp7 is thought to promote the formation of SGs, which resemble type-I SGs. NCp7 induces PKR activation, which leads to eIF2α phosphorylation, a known inducer of SG formation [342]. The NCp7-induced SG formation cannot be rescued by Gag expression and leads to the reduction of host cell protein translation. However, Staufen-1 is able to block this NCp7-induced SG formation by sequestering NCp7, and by binding to mRNA and stabilizing polysomes [306]. Staufen-1 has thus an effect on the early phase of infection by limiting the formation of SG by NCp7 and thus avoiding NCp7-induced cell translation arrest. Staufen-1 could also play a role in the late phase of infection, by modulating the balance between vRNA translation and packaging with the formation of specific granules (SHRNP).

Interestingly, several Gag partners mentioned in Table 1 are present in PBs, which are RNA granules found even in non-stressed cells. These granules contain proteins involved in mRNA decay and surveillance, such as the mRNA decapping enzymes DCP1 and 2 [343], the helicase DDX6 [344], DDX6 [345], FMRP1 [346] and eIF2 [347]. Most of these interactions are not attributed to NCp7 or the NC domain of Gag and will, thus, not be detailed here. However, they highlight the important contribution of RNA granules in the HIV-1 life cycle.

### 4.3. Viral Assembly

A number of other cellular factors interact with Gag and play various roles in virus production (Table 1). For instance, the tRNA^Lys^ synthetase interacts with the CA domain, helping the recruitment of tRNA^Lys^ into HIV-1 virions [278,279,280,281,282]. CypA interacts with the CA domain, modulating the maturation of virus particles [96,250,251,252,253] and the clathrin adaptor protein complexes AP-1, AP-2, and AP-3 interact with the MA domain of Gag, participating in Gag trafficking and virus release [234,235,236].

During the course of HIV-1 infection, Gag also interacts (via MA and NC domains) with Lyric [277]. Lyric for lysine-rich CEACAM-1–associated protein is also named AEG-1 (astrocyte elevated gene-1) or metadherin (metastasis adhesion protein MTDH). Lyric is a 64 kDa protein [348], with high expression in HIV-1 infected or gp120-treated primary human fetal astrocytes [349] and HIV-1 patient brains [350,351]. It is incorporated into viral particles and cleaved by the HIV-1 protease. This interaction seems to be conserved among retroviruses since Gag from equine infectious anemia virus (EIAV) and MLV interacts with endogenous Lyric. Mapping the domain of interaction indicated that MA and NC domains are important, in a membrane/RNA independent manner. However, co-immunoprecipitation of Lyric with Gag variants (NC domain substituted with a leucine zipper) or experiments with monomeric Gag demonstrate that NC-mediated multimerization is more important than NC itself for the interaction with Lyric, suggesting that MA may be the main contributor. All these data indicate a possible interaction of Lyric with the multimeric immature Gag lattice. The expression of the Gag binding domain of Lyric, encompassing amino acids 107 to 289, enhances Gag levels and viral infectivity. Furthermore, this domain overlaps with the binding domain of NF-kB and BCCIP, two proliferation/signaling proteins, and thus, possibly influences astrocyte inflammatory responses [277,350] and promotes neuroinflammation [350,351]. However, the precise function of the Gag-Lyric interaction is still not fully understood.

The cellular ATP-binding protein ABCE1 (also called HP68 or RNase L inhibitor), is another partner of Gag [217,218,352] that binds to the basic residues of the NC domain [217]. ABCE1 binds transiently, immediately after Gag translation but disassociates, when Gag processing starts [215,352]. Thus, ABCE1 plays a role in promoting virion formation by a post-translational mechanism [217,352]. ABCE1 is recruited to sites of assembly at the PM only in the presence of wild-type Gag but not with an assembly-defective Gag mutant [215,352]. Therefore, ABCE1 could be considered as a molecular chaperone that ensures Gag multimerization in an ordered kinetics mandatory for HIV-1 proper assembly [215,217].

A study from Mély’s group showed an interaction between Gag and the ribosomal protein L7 (RPL7), a protein involved in ribosome biogenesis and regulation of mRNA translation [296]. This interaction is dependent on the zinc fingers of the NC domain of Gag but independent from RNA binding, association of Gag with the PM, or Gag oligomerization. Also, the Gag-RPL7 complex drives the incorporation of RPL7 inside the virion. In addition, RPL7 stimulates the nucleic acid chaperone activity of Gag in vitro. Gag interacting with RPL7 could possibly act as a functional switch from RNA translation to Gag assembly.

As mentioned above, Gag interacts with Stau1, a protein involved in the transport and cellular localization of RNA [303,304,305]. This interaction takes place in the cytoplasm and at the PM, more specifically at cholesterol-enriched containing lipid rafts (acting as virus assembly microdomains). This binding involves the ds-RNA binding domain 3 (dsRBD3) of Stau1 and the NC domain of Gag (at least one of the two zinc fingers is needed) in an RNA dependent manner [303,304,307,353]. A ternary RNP complex comprising Gag, Stau1 and unspliced gRNA could be observed in cells [302,303]. Stau1 overexpression leads to vRNA encapsidation enhancement, which significantly affects the viral infectivity. Staufen1 is also incorporated in virions [353]. Using siRNA directed against Stau1 decreases HIV-1 infectivity [303]. Stau1 influences HIV-1 assembly by regulating Gag oligomerization at the PM [307]. A small region of 12 amino acids in the N-terminal domain of Stau1 is required for the Stau1-mediated enhancement of Gag multimerization [304]. Stau1 is also thought to influence the anterograde trafficking of Gag in cells [305]. Even though no specific binding domain of Gag was identified, EAP30 (an ESCRT II protein) interacts with Gag and Stau1, playing an important role in vRNA trafficking and gene expression. Indeed, siRNA against EAP30 reduces Gag and virion production while EAP30 overexpression leads to virus production increase. Moreover, KO of EAP30 results in vRNA accumulation in the nucleus [257].

HIV-1 Gag also interacts with Ubc9, an E2 SUMO-conjugating enzyme that post-translationally modifies target proteins and alters their function by the addition of SUMO. This interaction involves the NC-SP1-p6 domain [319], in line with the observation that the p6 domain possesses an Ubc9 binding site [165]. Gag and Ubc9 colocalize in perinuclear cytoplasmic clusters [273]. A similar result was observed for another retrovirus (Mason–Pfizer monkey virus), for which the full-length Gag also interacts with Ubc9 at a similar location in the cell [354]. Using siRNAs, it was shown that even though Gag synthesis, processing, or assembly into virions are not affected, Ubc9 is important for the production of infectious HIV-1 particles by influencing the stability and incorporation of mature Env into budding particles [319]. A model proposes that Gag trafficking modifications due to Ubc9 depletion could alter the Env–Gag interaction causing mature Env to be mistargeted for degradation before its transport to the PM [318].

The suppressor of cytokine signaling protein 1 (SOCS1), induced upon HIV-1 infection, also plays a role in the late stage of HIV-1 replication [300,301]. SOCS1 binds the MA and NC regions of the HIV-1 Gag polyprotein via its central SH2 domain and enhances the stability and trafficking of Gag from perinuclear clusters to the PM via a proper microtubule network [300]. Furthermore, depletion of SOCS1 in cells enhances the lysosomal degradation of Gag. Therefore, SOCS1 could be considered as a positive regulator of Gag trafficking/stability needed for the efficient production of virions via an interferon signaling-independent mechanism [301].

The involvement of the cytoskeleton in the HIV-cell cycle was also investigated. Early studies indicated that several cytoskeletal proteins (e.g., actin, cofilin, and moesin) are present in HIV-1 virions [25,220,355]. A direct interaction between F-Actin and Gag via its NC domain was evidenced using multiples techniques [160,219,220,221]. However, ex vivo colocalization experiments using immunofluorescence or GFP tagged proteins were not really convincing [221,356,357]. Moreover, Gladnikoff et al. [358] found that expression of a Gag Leucine Zipper chimera (noted GagLZ where NC domain was replaced by LZ from the *S. cerevisiae* transcription factor GCN4) resulted in a slower budding of VLPs, and the absence of actin remodeling; confirming the importance of NC domain in binding actin. Moreover, a star-shaped actin filament emanating from the budding site was observed [358]. Furthermore, cryoEM tomography confirmed the presence of filamentous actin in the vicinity of viral budding sites often in contact with the Gag layer [359]. Treating HIV-1 infected cells with cytochalasin D (an F-Actin disrupting agent), or wortmannin (inhibitor of myosin light chain kinase) affected HIV-1 budding [357]. However, another paper argued against a specific recruitment of actin through NC at the budding site, since actin was found in equal quantity in VLP obtained from wild type (WT) Gag or GagLZ and minor effect on assembly rates was observed. Depending on the cell type, a number of 11 to 125 actin molecules per virion was deduced [223], clearly less than in a previous estimation (around 250 actin/virion) [25]. Therefore, it is speculated that actin is not specifically uptaken from the cytosol and that the putative Gag-NC-Actin complex does not play a major role in HIV-1 assembly [223].

A major cytoskeleton regulator IQ motif-containing GTPase activating protein 1 (IQGAP1) ubiquitously expressed in many cell types (including HIV-1 target cells) interacts with the NC and p6 domains of Gag in an RNA independent manner [272]. The interaction does not depend on the ability of Gag to interact with the membrane, but it specifically plays a role during the late stage of infection. Furthermore, IQGAP1 is present in HIV-1 virions produced from human monocyte-derived macrophages [26]. While overexpression of IQGAP1 diminishes the viral progeny, its knockdown leads to increased viral production, demonstrating the negative regulation of IQGAP-1 by preventing Gag-PM accumulation [272]. In comparison, its interaction with Gag from the Moloney murine leukemia virus (MMuLV), through the MA domain, regulates virus replication positively, playing dual roles in the early and late phase of infection [360].

The insulin-like growth factor II mRNA binding protein 1 (IMP1), an evolutionary regulatory protein involved in RNA transport and translation, interacts via its KH3 and KH4 domains (hnRNP K homology) with the NC domain of Gag at the rim of the cell [270]. IMP1 is packaged into virus particles. Overexpression of wild-type IMP1 but not of Gag-binding deficient IMP1, interferes with HIV-1 assembly by inhibiting vRNA packaging and blocking Gag cleavage. This impedes virus maturation, leading to the accumulation of immature virions at the cell surface and a reduction of virion infectivity [270]. IMP1 is also found in Stau1-HIV-1-dependent RNPs [305]. IMP1 binds also to Rev, altering Rev function in vRNA expression by promoting the accumulation of multiple spliced HIV-1 RNA [361]. Additionally, overexpression of IMP1 with eGFP enhances its effect on the HIV-1 viral cycle, making it a better tool to block HIV infection [270,361].

Finally, the drosophila discs large protein (Dlg1/hDlg/SAP97), another negative regulator targeting a very late stage of infection, was described [256]. Dlg1 is a membrane associated guanylate kinase playing a role of a scaffold protein at the PM. Dlg1 directly interacts with Gag both in vitro and in vivo. However, no incorporation in the virions was reported. The NC domain of Gag is mandatory for this interaction, but it does not involve RNA. The knockdown of Dgl1 enhances HIV-1 infectivity but there is no effect on Gag synthesis or virus release. Surprisingly, a higher quantity of Env protein in cells and virions, as well as Gag and Env redistribution at specific sites were observed [256].

Thus, Dlg1, IQGAP1 or IMP1 are Gag-binding proteins that negatively regulate the late stage of infection, making them promising targets to block HIV-1 infection.

### 4.4. Viral Budding

The viral budding and release of nascent viral particles are mainly driven by the p6 domain of Gag. However, the NC domain is essential for this process by favoring the recruitment of the ESCRT machinery and by interacting with different cellular proteins that increase the virus release efficacy.

Transmission EM images of 293T, HeLa and T cells transfected with HIV-1 coding plasmid (pNL4-3) have shown that the Gag mutants in which the NC domain was replaced by a leucine zipper (conserving the gag multimerization capacity) form immature budding particles that remain attached to the cell surface in a similar way to those observed for L-domains mutants. Interestingly, the budding defects of NC mutants can be rescued by ectopic expression of Nedd4.2, an ubiquitin E3 ligase which binds to Gag and recruits the ESCRT III members, or by expression in trans of Gag carrying NC domain but lacking all L-domains. Thus, the NC domain is essential for the recruitment of ESCRT members by Gag [316]. In addition, Popova et al. [362] have shown that replacement of the entire NC-SP2-p6 region by LZ leads to an alternative ESCRT-independent mechanism for HIV-1 particle production. This suggests that the NC-SP2 region confers the dependence of the HIV-1 viral release on the ESCRT machinery [362]. Mechanistic studies further reveal that the NC domain plays an important role in both PTAP/Tsg101 and LYPXnL/ALIX mediated release pathways. The function of L-domains depends on their location within the Gag polyprotein, which implies that they cooperate with other Gag regions in order to optimize their efficacy. When p6 PTAP motif was inserted into Gag of other retroviruses, these mutants conserved their release activity if the HIV-1 NC was associated with p6 or if the PTAP sequence was inserted in proximity to the NC [363,364]. These results indicate that the NC domain probably plays a role in PTAP/Tsg101 binding. Even though co-IP experiments [227] indicated that mutant HIV-1 lacking the NC domain retains the ability to bind Tsg101, other reports showed that Tsg101 binding to the PTAP L-domain is NC-dependent [317,365].

In the work of ElMeshri et al. [317] confocal microscopy observations revealed that the expression of Gag in HeLa cells expressing fluorescently labeled Tsg101 leads to the delocalization of the latter from the cytosol to the PM. Their interaction was confirmed by FRET/FLIM measurements. Further analyzes showed that Gag mutants with deleted NC domain or ZF impaired the Gag-induced Tsg101 delocalization and Tsg101/Gag binding, while mutants with at least one ZF retained the same phenotype as WT Gag. Furthermore, the binding interface between Tsg101 and NCp15 was identified by NMR chemical shift mapping. The contact region includes the PTAP sequence in p6 and several amino acids in the ZFs and C-terminal portion of the NC domain [317], suggesting that NC cooperates with p6 in the recruitment of Tsg101 and thus, in favoring HIV-1 release. Chamontin et al. have further characterized the role of the second ZF (ZF2) of NC in the recruitment of Tsg101 [365]. They showed that ZF2 deletion impairs the Gag/Tsg101 interaction at the PM. In addition, electron micrographs of cells expressing ZF2-deleted Gag mutants show aberrant budding structures and immature particles attached to the PM. Released viral particles contain lower amounts of Tsg101 compared to WT virions. The budding phenotype and Tsg101 incorporation into the VLPs are restored by trans-complementation of Tsg101. Interestingly, deletion of ZF2 activates late RTion generating viruses with high DNA content. Altogether, this study clearly shows a role of the NC domain in the Gag/Tsg101 interaction and the control of RTion timing during HIV-1 replication.

As for the PTAP L-domain, the function of the LYPXnL motif depends on the NC domain of Gag [227,229,231]. The binding of ALIX and its isolated Bro1 domain to the NC is mediated by the positively charged residues in the NC ZF motifs, but its dependence on NAs is not clearly established. Popov et al. have shown that the binding of Bro1 domain to NC is resistant to nuclease treatment [229] while this treatment abrogates the capture of Bro1 by GST-NC-SP2-p6 in another report [231]. This divergence is probably related to the p6 fragment in the former experiment or to differences in experimental protocols. Nevertheless, the implication of RNAs in NC Bro1 binding seems plausible since mutations of NC basic residues (known for their role in RNA binding) also eliminate NC interaction with Bro1 [231]. Noteworthy, Bro domains of other proteins such as Brox, HP-PTP and Rhodophilin 2 are also recognized by NC, but their possible role in HIV-1 release is debated [227,228]. 

The interaction between ALIX and the NC domain of Gag has been confirmed in the cellular context by experiments showing that overexpressed ALIX is incorporated into VLPs by WT Gag, but not by a Gag mutant where the NC domain is replaced by a LZ. Moreover, the ALIX/LYPXnP-mediated rescue of the HIV-1Δ_PTAP_ mutant budding phenotype is dependent on the Gag NC domain [227,229]. This rescue is abolished by C_28,49_S and Δ15–39 mutations of the NC domain, which prevent its binding to ALIX. In addition, Dussupt et al. [227] have shown that over-expression of ALIX Bro1 domain, but not of full-length ALIX rescues the release of an HIV-1 mutant lacking both PTAP and LYPXnP domains. This rescue depends on the NC binding to Bro1 which is likely needed to link Gag to the ESCRT III complex [227]. Interestingly, Sette et al. [230] have found some similarities between ALIX interaction with NC and its interaction with the PDZ domain of its native partner syntenin that controls the association of syntenin to the PM. The authors raised the hypothesis that NC role in ALIX-mediated viral egress is to bind to lipid membranes and drive the ALIX Bro1/ESCRTs complexes to cholesterol-rich domains that favor virus assembly [230]. In conclusion, all these studies show that the NC domain is essential for the recruitment of the ESCRT machinery by both L domains of Gag. NC presumably cooperates with the PTAP domain in the recruitment of ESCRT proteins and plays a role in the binding of the ALIX/Bro1 domain with ESCRT-III required for LYPXnL-mediated budding.

The role of NC in virus budding seems to be conserved in other lentiviruses (SIVcpzGAB2 and SIVsmmE543) whose release depends on interactions of PTAP and LyPXnL L-domains with Tsg101 and ALIX, respectively. Similarly, a functional NC domain has been shown to condition the viral release of EIAV that relies solely on ALIX binding to the LYPXnL domain [366].

The NC domain of Gag is also involved in alternative recruitment of the ESCRT machinery, via ubiquitination. Ubiquitination is a regulation mechanism of the protein transport between different intracellular vesicular compartments. Several ubiquitin ligases play a role in HIV-1 budding and release. Nedd4-like ubiquitin ligase family is recruited by many retroviruses via their PPPY-type L-domain [367,368]. The Nedd4-like family ubiquitinates cargo proteins and targets them into the MVBs sorting pathway. In the viral context, Nedd4 ligases connect Gag to ESCRT-III and VPS4 that drive the viral egress. Although HIV-1 Gag does not present a PPPY sequence, two Nedd4 E3 ubiquitin ligases, Nedd4-1 and Nedd4-2, co-immunoprecipitate with Gag and their overexpression can rescue the budding phenotype of HIV-1∆_PTAP_ mutants. In the case of Nedd4-1, this rescue occurs via the ALIX/LYPXnL pathway and requires the basic residues in the NC domain [286], while Nedd4-2 ubiquitinates the Gag protein and rescues the viral release of HIV-1 independently of both L-domains and NC domain [287,288,369].

The NC domain of Gag also plays a role in the activation of the soluble N-ethylmaleimide-sensitive factor attachment protein receptor (SNARE) machinery. The soluble SNARE proteins regulate several membrane remodeling events involved in membrane vesicle trafficking and cytokinesis. In 293T cells, the siRNA knock-down of NSF, a critical SNARE component, leads to disruption of Gag processing and HIV-1 virus release. The same phenotype is observed by expressing a dominant-negative NSF-DN mutant, defective in ATP hydrolysis. The viral target of NSF seems to be the NC domain since NC-lacking, but not MA-lacking Gag mutants are insensitive to SNARE perturbations. In CD4^+^/CXCR4^+^/CCR5^+^ HeLa cells, the expression of NSF-DN perturbs the binding of WT Gag as well as of MA-deficient and HIV-1∆_PTAP_ Gag mutants to the PM. Taken together, these results indicate that the SNARE machinery perturbs the binding of Gag to the PM via its NC domain, which leads to defects in virus release [299,370].

Other cellular proteins, notably several RNA binding and ribosomal proteins, likely intervene in NC-dependent events in late budding and release of HIV-1 viruses [290]. Among them, nucleolin is of particular interest because of its known implication in the late phases of MLV replication [289]. Nucleolin is a non-ribosomal protein mainly present in the cell nucleolus where it assists the ribosomal biogenesis and nucleo-cytoplasmic transport [371]. A small fraction of nucleolin is also found in the cytoplasm and at the PM. The binding of nucleolin to HIV-1 Gag has been shown by co-IP and yeast two-hybrid screen [289,290,291]. In a similar way to MLV, these interactions are dependent on the NC domain and RNAs. However, unlike MLV for which the nucleolin inhibits the virion release, this protein enhances particle assembly and release of HIV-1 virus. Analysis of the budding of Gag proteins expressed by a recombinant vaccinia virus or an HIV-1 provirus reveals that overexpression of nucleolin enhances the level p24 in the cell supernatant and that this increase is even higher in the presence of Psi packaging signal. In addition, when expressed in rabbit kidney RK3 cells, human nucleolin is incorporated with the gRNA into the VLPs and this incorporation is significantly enhanced when gRNA bears the Psi signal. Interestingly, the HIV-1 viral particles containing nucleolin also show an increased infectivity [291]. Noteworthy, nucleolin incorporation into VLPs was not observed in COS7 cells expressing the HIV-1 provirus [289]. In summary, nucleolin may play a role related to gRNA binding by the NC domain of Gag and its incorporation into the nascent virions during the assembly process.

## 5. Conclusions

This review highlights the multiple roles of the structural protein Gag in HIV-1 replication, through specific interactions with cellular components, notably during the late phase of the replication (Table 1). We focused on the NC domain of Gag and its interactions with cellular partners that are mandatory at different steps of the HIV-1 replication cycle (Figure 2). Furthermore, as the NC domain is a highly conserved region [372], it represents an ideal target to develop compounds inhibiting HIV-1 and, more specifically, viral assembly. Several inhibitors have been studied over the past few years and their potential therapeutic effects against HIV-1 have been recently reviewed [373,374]. Targeting the late phase of the HIV-1 cell cycle and especially the Gag-cellular proteins interactome represents a promising challenge for the development of new drugs.

## Figures and Tables

**Table 1:** Cellular proteins interacting with HIV-1 Gag polyprotein.

Partner of Interaction	Classification of the Protein	Role of the Protein	Gag Interaction Domain(s)	Role of the Protein or the Interaction in the Replication Cycle	Methods Used to Demonstrate the Interaction	References
ABCE1 (ATP-binding cassette sub-family E member 1) = HP68	ATPase, member of the ATP-binding cassette (ABC) transporters superfamily and ATP-binding cassette, sub-family E (OABP)	Inhibits the action of ribonuclease L (which presents antiviral activities) and has an effect on tumor cell proliferation and anti-apoptosis	NC	Essential for post-translational events in immature capsid assembly, Recruits a protein interfering with the late steps of the replication cycle	Radiolabeling Co-IP	[215,216,217,218]
Actin	Family of globular multi-functional proteins, forming microfilaments Component of the cytoskeleton and of the contractile apparatus in muscle cells	Essential for muscle contraction, cell mobility and remodeling, cell division and cytokinesis, intracellular trafficking and cell division	NC	Could play a role in assembly and/or other steps of the replication cycle	Co-IP in vitro protein binding assay Co-Fractionation	[160,219,220]
			Gag	Viral budding	Intensity-based FRET Co-Fractionation in vitro protein binding assay	[221,222,223]
			MA	Involved in proper localization and activation of the Reverse Transcription complex	Co-IP	[41]
ADAR1 (adenosine deaminase acting on RNA1)	Adenosine deaminase	Catalyzes the conversion of adenosine to inosine within a dsRNA	nd	Incorporated into particles, role needs to be elucidated	Dual tag affinity purification IP	[224]
Angiomotin AMOT (also AMOTL1, AMOTL2)	Motin family member (also angiomotin-like protein)	Functions related to endothelial cell migration, angiogenesis, embryonic cell movements, and maintenance of cell polarity	nd	Links Gag and NEDD4L, promotes HIV release from infected cells. Helps to complete immature virion assembly prior to budding.	Pull down experiment	[225]
AGO2 (Argonaute-2)	Active part of RNA-induced silencing complex (RISC)	Necessary for RNA-mediated gene silencing (RNAi) by the RISC	nd	Probably involved in capsid assembly (this function is independent of miRNA and translation regulation)	Co-IP	[226]
ALIX (apoptosis-linked gene 2-interacting protein X) = AIP1	Adaptor protein	Involved in endocytosis, multivesicular body biogenesis, membrane repair, cytokinesis, apoptosis and maintenance of tight junction integrity	NC and p6	Recruits ESCRT proteins for the release of newly formed viral particles	GST pull-down	[190,191,227,228,229,230,231]
Anx2 (annexin II)	Calcium-dependent phospholipid-binding protein	Involved in exocytosis, endocytosis, membrane organization, linkage of the F-actin cytoskeleton to the PM fibrinolysis…	nd	Increases Gag processing and virus production	Co-IP BiFC In vitro protein binding assay, Co-IP	[232,233]
AP-1 (clathrin-associated adaptor protein 1)	Clathrin adaptor proteins	Involved in intracellular transport of lysosomal hydrolases between the trans-Golgi network, and endosomes	MA	Transports Gag to the sites of active budding, facilitates Gag interactions with other cellular proteins	Yeast two-hybrid GST pull down Co-IP	[234]
AP-2 (clathrin-associated adaptor protein 2)		Works on the cell membrane to internalize cargos in clathrin-mediated endocytosis	MA-CA junction	AP-2 complex plays a role in the regulation of the virus assembly/release	GST pull-down In-vitro SPR measurements	[235]
AP-3 (clathrin-associated adaptor protein 3)		Responsible for the transport of proteins to lysosomes and other organelles	MA	Directs Gag trafficking to MVB and participates in virus assembly	Yeast two-hybrid GST pull-down Co-IP	[236]
APC (adenomatous polyposis coli protein)	Tumor suppressor	Suppresses Wnt signaling Implicated in cell adhesion and migration	MA HBR domain	Promotes Gag multimerization at the PM, vRNA incorporation and targeting at virological synapses	TAP- tag/MS screen GST pull-down Co-IP with endogenous protein	[237]
aPKC (atypical protein kinase C)	Serine/Threonine kinase	Kinase implicated in cell polarity and migration	nd	aPKC regulates HIV-1 infection via the phosphorylation of Gag-p6 which enhances the incorporation of Vpr into virions	Luminescent proximity assay Co-IP	[238]
APOBEC3G (A3G) APOBEC3F (A3F)	RNA/DNA cytidine deaminase-editing enzyme	Innate antiviral immunity	NC	Cellular HIV restriction factor Once incorporated in virions causes deamination of nascent DNA during RT	BiFC GST pull-down Co-IP	[161,239,240,241]
ApoH (apolipoprotein H)	Apolipoprotein component of circulating plasma lipoproteins	Beta-2-glycoprotein I Component of circulating plasma lipoproteins	MA	nd	Capture of sera virus Immunoblot with MA	[242]
BAF (barrier-to-autointegration factor)	Chromatin protein	Potential roles in cell division, but no precise function defined	MA	Potential role in PIC assembly and structure	Co-IP	[243]
CaM (calmodulin)	Ubiquitous multifunctional calcium-binding messenger	Implicated in numerous pathways, such as inflammation, metabolism, apopotosis, immune response	MA	Binding leads to MA myristate exposure Binding may induce MA transient unfolding Binds also Gp41	Isothermal titration calorimetry Mass spectrometry Gel filtration	[244,245,246]
CD81, CD82	Tetraspanins	Membrane glycoproteins involved in cell-cell adhesion, fusion, signal transduction, proliferation and differentiation	nd	Facilitates the viral egress	Co-IP	[247]
Citron kinase	Ser/Thr protein kinase	Effector of RhoA GTPase Implicated in mitosis and DNA damage control	nd	Enhances virus production by promoting Gag ubiquitination Induces viral release via the MVB pathway	Co-IP	[248]
CNP (2′,3′-cyclic-nucleotide 3′-phosphodiesterase)	Membrane-associated enzyme	2H phosphoesterase superfamily	MA	Inhibits particle formation at the PM	GST pull-down with crosslinking Co-IP	[249]
CypA and CypB (cyclophilin A and B)	Peptidyl-prolyl *cis-trans* isomerase	Facilitates protein folding and trafficking CypA is involved in T-cell activation	CA	Incorporated into virions, Protects HIV-1 from restriction factor TRIM5α	Yeast two-hybrid GST pull-down Co-IP	[96,250,251,252,253]
DCP1 and DCP2 (decapping protein)	mRNA decapping enzymes	Component of mRNA decapping complex with DDX6 Role in 5′-to-3′ RNA degradation pathway	nd	May be a component of HIV-1 assembly intermediates	Co-IP with overexpression	[226,254]
DDX6 (DEAD-box RNA helicase 6)	RNA helicase	Role in mRNA decapping and degradation	nd	Facilitates capsid assembly	Co-IP	[254]
DDX17 (DEAD-box RNA helicase17)	RNA helicase	Role in RNA metabolism	nd	Modulates HIV RNA metabolism May promote infectious particle production	Proximity biotinylation assay Co-IP	[36,255]
DLG1 (discs large homolog 1) = SAP97)	Membrane-associated guanylate kinase (MAGUK) family	Scaffold, anchor and adaptor protein at the PM	NC	RNA independent interaction Restricts HIV infectivity Modulates Gag cellular distribution	Co-IP with endogenous DLG1 GST-pull down	[256]
EAP30 (ELL associated protein of 30 kDa)	ESCRT-II	Roles of ESCRT-II in mRNA trafficking and in promotion of the inward budding of vesicles from the membranes of late endosomes	nd	EAP30-Gag-Staufen1 are part of the HIV-1 RNP, promoting the trafficking of HIV-1 Gag and the vRNA to directly influence viral production	Co-IP TriFC	[257]
eEF1α (eukaryotic elongation factor 1-alpha)	Translation elongation factor	Responsible for the enzymatic delivery of aminoacyl tRNA to the ribosome	MA(HBR) and NC	May contribute to tRNA incorporation into virions and plays a role in viral uncoating Interacts with RT	Yeast two-hybrid GST pull-down Co-IP	[258,259]
eEF2 (eukaryotic elongation factor 2)	Translation elongation factor	Promotes GTP-dependent translocation of the ribosome	CA	Required in assembly blockade of Gag-mediated stress granules	Mass spectrometry Co-IP with endogenous protein	[260]
Filamin A	Filamin	Promotes orthogonal branching of actin filament and links them to membrane glycoproteins	CA	Involved in Gag trafficking to the PM	Yeast two-hybrid GST pull-down Co-IP	[261]
FMRP1 (fragile X mental retardation protein)	mRNA binding protein	Part of neuronal granules May participate to RNA transport and expression regulation	NC	RNA independent interaction Incorporated in virions May regulate HIV infectivity and modulate RNP packaging into virions	Co-IP with endogenous protein	[262]
GAPDH (glyceraldehyde 3-phosphate dehydrogenase)	Oxidoreductase	Enzyme implicated in glycolysis	MA and CA	Incorporated in virions Negatively regulates infection	Co-IP Yeast two-hybrid	[263]
HEED (human polycomb protein EED)	WD-40 repeat and Polycomb-group protein families	Involved in maintaining the transcriptional repressive state of genes	MA	Negative effect at early phase of infection Binds also IN and Nef	Yeast two-hybrid GST pull-down	[264]
eIF5B (eukaryotic initiation factor 5 B) = eIF2	Translation initiation factor	Mediates the joining of ribosome 60S and 40S subunits	MA	MA inhibits eIF5B mediated translation	Yeast two-hybrid GST pull-down Co-IP	[265]
HARS2 (histidyl-tRNA synthetase homolog) = HO3)	Aminoacyl-tRNA synthetase	Catalyzes the ATP-dependent ligation of histidine to the 3′-end of its cognate tRNA	MA	Enhances infectivity Found in virions	Yeast two-hybrid Co-IP	[266]
ICAM-1 (intercellular adhesion molecule A) = CD54	Immunoglobulin superfamily	Endothelial- and leucocyte-associated protein Mediates cell -cell adhesion	MA	Promotes HIV-mediated syncitia formation Important for binding of HIV-infected dendritic cells to CD4+ T cells ICAM-1 promotes HIV entry into cells	Virus immunocapture assay	[267,268]
IMP1 (insulin-like growth factor II mRNA binding protein 1)	RNA binding factor	Recruits target transcripts to cytoplasmic protein-RNA complexes (mRNPs)	NC (2nd zinc finger)	Blocks the formation of infectious particles	Co-IP	[269,270]
IP3R (1,4,5-inositol trisphosphate receptor)	Ca^2+^ signaling protein	Functions as Ca^2+^ ion-specific channel on the membrane of the endoplasmic reticulum (ER)	nd, potentially p6	Gag modulates both ER Ca^2+^ release and refilling via its PTAP domain	Co-IP close proximity experiments: IEM (immunoelectron microscopy) TIRF	[271]
IQGAP1 (IQ motif-containing GTPase activating protein)	Scaffold protein	Regulator of many cellular processes (vesicle trafficking, endocytosis…), - cytoskeleton regulator affecting both microtubules and actin	NC and p6	Negative regulator: factor inhibiting efficient budding by preventing the accumulation of Gag at the cellular PM	Co-IP	[272]
KIF4 (kinesin superfamily protein)	microtubule (MT)-stimulated ATPase in the kinesin motor family	KIF4 regulates the movement of multiple intracellular components, implicated in chromosome segregation during mitosis, and cytokinesis as well as in the regulation of programmed cell death in juvenile neurons.	MA	Regulates Gag trafficking and stability	Yeast two-hybrid Co-IP	[273,274,275]
LC3 = Atg8 (autophagy-related protein 8)	Autophagy factor	Facilitates autophagosome biogenesis and wrapping around autophagic targets	nd	Increases Gag processing and HIV yields	Co-IP	[276]
Lyric (lysine-rich carcinoembryonic antigen-related cell adhesion molecule coisolated) = astrocyte-elevated gene 1EG-1 or metadherin	Adhesion molecule	Implicated in various signaling pathways, suggested to have anti-apoptotic effects and to be involved in tumorigenesis	MA and NC	Potential role in regulating infectivity Implicated in HIV-1 associated neuropathy and potentially promoting HIV replication	Affinity purification Co-IP	[37,277]
LysRS (lysyl-tRNA synthetase)	Lysyl-tRNA synthetase	Catalyzes the formation of Lys-tRNA	CA (Helix 4 of the C-terminal domain)	Packaging of tRNA^Lys^ into the virions	GST pull-down Fluorescence anisotropy FPLC Circular dichroism molecular dynamics	[278,279,280,281,282]
MAP1A and MAP1S (microtubule-associated proteins)	Microtubule-associated protein family	Regulate the stability and the dynamics of microtubules, guide the microtubules to a specific location, mediate interactions with cellular proteins	CA	Promote HIV trafficking to the nucleus, help tether viral capsids to microtubules	Yeast two-hybrid Capsid-binding assay Proximity ligation assay	[97]
MAPK/ERK-2 (mitogen-activated protein kinase/extracellular signal-regulated kinase 2)	Serine–threonine kinases	Involved in regulation of meiosis, mitosis, and post-mitotic functions in differentiated cells	CA (Proline residues)	Interaction results in Gag incorporation into virus particles and may be essential for retroviral replication	Co-IP GST pull-down	[283]
MOV10 (Moloney leukemia virus 10 homolog)	Super family-1 RNA helicase	Type I interferon stimulating gene Plays a role in miRNA-mediated regulation Broad antiviral activity	NC	Incorporated into virions Inhibitory effect on HIV infection	GST pull-down Co-IP	[284,285]
Nedd4-1 (neural precursor cell expressed developmentally down-regulated protein 4) and Nedd4-2	E3 ubiquitin-protein ligase	Ubiquitinate and target cargo proteins into the MVB sorting pathway	p6 and NC	Stimulate viral release by ubiquitination of Gag leading to the recruitment of ESCRT complexes	Co-IP Yeast two-hybrid Incorporation of Nedd4-2 into VLPs	[286,287,288]
Nucleolin	RNA binding protein	Major non-ribosomal nucleolar proteins nucleolus, Role in ribosome biogenesis	NC	Enhances virion assembly and release	Yeast two-hybrid Co-IP Tandem affinity purification	[289,290,291]
PACSIN2 (protein kinase C and casein kinase substrate in neurons 2)	F-BAR domain family	Implicated in remodeling membrane and actin cytoskeleton	p6	Promotes cell-to-cell transmission, enhancing HIV-1 spreading (connecting Gag to actin?)	Co-IP	[292]
PDZD8 (PDZ domain-containing protein 8)	Cytoskeletal regulatory protein, ER membrane protein	Plays a role in the regulation of cell morphology, cytoskeletal organization and endosomal maturation	CA	Positive mediator of retroviral infection, promoting early stage of infection by stabilizing CA to support HIV-1 infection	Yeast two-hybrid Co-IP	[293,294,295]
RPL7 (ribosomal protein large 7)	Ribosomal protein	Involved in ribosome biogenesis and regulation of mRNA translation	NC (zinc fingers)	Promotes Gag chaperone activity	Yeast two-hybrid Co-IP	[296]
RVB-2 (RuvB-like 2)	AAA+ superfamily member	Multifunctional protein involved in DNA repair, nonsense-mediated mRNA decay, humoral immunity regulator …	MA	Role in controlling viral protein expression (Env and Gag)	Tandem affinity purification Co-IP	[297,298]
RPS6 (ribosomal protein small 6)	Ribosomal protein	Ribosome biogenesis	nd	nd	Proximity-dependent biotin identification (BioID) Co-IP	[36]
SNARE (soluble N-ethylmaleimide-sensitive factor attachment protein receptor)	Soluble NSF Attachment protein receptor family	Mediates vesicle fusion	NC	Role in assembly and release, likely by affecting cellular trafficking pathways required for Gag transport and association with the PM	In vitro protein binding	[299]
SOCS1 (suppressor of cytokine signaling protein 1)	Suppressor of cytokine signaling (SOCS) family	Takes part in a negative feedback loop to attenuate cytokine signaling	MA and NC	Regulates positively late stages of HIV-1 infection by facilitating Gag intracellular trafficking to the PM and its stability via the microtubule network which may as well enhance Gag ubiquitination	GST-pull down Co-IP	[300,301]
Staufen-1 (dsRNA-binding Staufen homolog 1)	dsRNA-binding proteins family	Involved in the transport and/or localization of mRNAs to different subcellular compartments and/or organelles	NC	Part of RNP complex (Gag+vRNA+Staufen-1 +other proteins). Participates in HIV-1 assembly by influencing Gag multimerization, and in the intracellular trafficking of Gag during viral egress. Staufen1 also plays important rescue roles (vRNA translation…) during cellular stress.	Co-IP BRET BiFC Tandem affinity purification TriFC	[257,302,303,304,305,306,307,308]
TIP47 (tail-interacting protein of 47bkDa) = M6PRBP1 (mannose-6-phosphate binding protein)	Peripilin protein family	Involved in the endosome-to-TGN retrograde transport of mannose-6 phosphate receptors	MA	Involved in the incorporation of HIV-1 Env into HIV-1 Gag particle during viral assembly (T-cell and macrophage)	Yeast two-hybrid GST-pull down Co-IP NMR Surface plasmon resonance (SPR) binding assay	[309,310,311]
TRIM5α (tripartite motif-containing protein 5)	TRIM (tripartite motif) protein family	Retrovirus restriction factor, mediates species-specific, early block to retrovirus infection	CA	Mediator of innate cellular resistance to infection acting on the capsid; cellular factor blocking virus production by actively degrading viral Gag polyproteins	Trim5α restriction assay	[312,313,314,315]
Tsg101 (tumor susceptibility gene 101)	VPS (vacuolar protein sorting) family, component of ESCRT I complex	Regulates the vesicular trafficking. Involved in sorting of cargos into MVBs. Required for cytokinesis, plays a role in cell growth and differentiation and acts as a negative growth regulator	p6 and NC	The binding to p6 leads to the recruitment of ESCRT proteins and the following viral release	GST pull-down, Yeast two-hybrid Co-IPFRET-FLIM NMR chemical shift mapping	[164,179,316,317]
Ubc9 (ubiquitin carrier protein 9)	E2 SUMO-1 conjugating enzyme	Post-translationally modifies target proteins and alters their function by SUMOylation	NC-p6	Plays a role in the production of infectious HIV-1 virions, influencing the stability and trafficking of Env proteins to the site of assembly	GST-pull down Yeast two-hybrid Co-IP	[165,318,319]
UBP (U-binding protein)	TPR (tetratricopeptide repeat) family of proteins	TPR family: organelle-targeting proteins, proteins involved in mitosis, immunophilins and nuclear phosphatases	nd	Intermediary between Vpu and Gag and likely plays a role in virus assembly or release	Yeast two-hybrid in vitro protein binding assay	[320]
UPF1 (upframeshift protein 1) = UPF3B	ATP-dependent RNA helicase of the SFI superfamily	Required for nonsense-mediated mRNA decay in eukaryotes (RNA stability). Also involved in DNA repair, cell cycle progression, DNA replication, telomere metabolism	nd	Part of the Staufen-1 RNP complex in the cytoplasm. Role in the maintenance of HIV-1 RNA stability and protein synthesis	Co-IP Tandem affinity purification assay	[308,321,322]
VAN (virion-associated nuclear shuttling protein)	Nuclear/cytoplasm shuttling protein	unknown	MA	Role during early phase of replication. Potentially facilitating nuclear import and retention of the PIC.	Yeast two-hybrid GST pull down	[323]

BiFC: Bimolecular Fluorescence Complementation; BRET: Bioluminescence Resonance Energy Transfer; Co-IP: Co-Immunoprecipitation; FLIM: Fluorescence Lifetime Imaging Microscopy; FRET: Fluorescence Resonance Energy Transfer; GST: Glutathione-S-transferase; IEM: Immuno-Electron Microscopy; MVB: MultiVesicular Bodies; nd: not determined; SPR: Surface plasmon resonance; TAP: Tandem Affinity Purification; TIRF: Total Internal Reflection fluorescence.
